# Demands for Community Services and Associated Factors among Residents in Smart Communities: A Case Study of Xuzhou City

**DOI:** 10.3390/ijerph20043750

**Published:** 2023-02-20

**Authors:** Jiongxun Chen, Linxiu Wang, Tiantian Gu, Chenyang Wang, Enyang Hao

**Affiliations:** School of Mechanics and Civil Engineering, China University of Mining and Technology, Xuzhou 221116, China

**Keywords:** smart community, residents’ demands, influencing factors, binary logistic regression

## Abstract

Smart community enables a sustainable and livable community future, in which residents’ demands play an important role in its success. Though great efforts have been made to encourage residents’ participation in the implementation of smart communities, inefficient service supply still exists. Thus, this study aimed to classify residents’ demands for community services in smart communities and to explore relevant influencing factors based on the developed conceptual framework. Data from 221 respondents in Xuzhou city of China were analyzed by using binary logistic regression. The results indicated that more than 70% of respondents had demands for all community services in smart communities. Moreover, the demands were influenced by distinct factors, including sociodemographic characteristics, living characteristics, economic characteristics, and individual attitude characteristics. The types of community services in smart communities are clarified and fresh insights are provided into associated factors related to residents’ demands for these services in this study, through which enhanced provision of community services and effective implementation of smart communities can be achieved.

## 1. Introduction

Smart community is generally considered the micro unit of the smart city that provides convenient services for residents through new information technology [1]. It has been nearly 30 years since an initiative for building a “smart community” was first launched by San Diego State University’s International Center for Communication in 1992. With the proposal of the concept of the “smart city” from the International Business Machines Corporation (IBM) of the United States in 2009, implementing smart communities started to be in full swing in the world. Hence, information technology is gradually being incorporated into the implementation of communities. For example, various facilities using advanced Internet technology have been utilized in the implementation of the community to enhance the quality of public services [2,3,4]. Meanwhile, residents’ demands have received growing attention, and the community begins to shift from intelligent to smart, which integrates residents’ demands into the development to create a more comfortable environment for them.

Compared to other countries, a relatively slow pace has been observed in China when it comes to implementing smart communities. The government in the early stage focused on how to effectively apply Internet technology to the community [5,6]. With the continuous advancement of the smart community, the government began to realize the importance of residents’ participation in the development of the community and to recognize that residents’ demands are of great significance for the provision of community services. For instance, Smart Community Construction and Operation Guide (2021), jointly issued by the State Information Center Smarter City Development and Research Center and Ruicity Digital Technology Company Limited in October 2021, proposed that the community was essential for residents’ daily lives, and residents in the community had different needs when it comes to community services [1]. There will be serious consequences if residents’ needs are not addressed. For example, ineffective supplies of services in communities were caused due to the lack of consideration for residents’ demands, such as unreasonable road planning and sewage treatment ponds being placed in the wrong locations in a community of Fenglou Village in Zhaoqing City, Guangdong Province of China, which resulted in residents’ dissatisfaction [7]. Therefore, residents’ diverse demands for community services should be taken into account [1].

Previously, smart communities have been studied primarily from the aspects of advanced computer technologies [8,9,10,11,12,13,14,15], design of operating modes [16,17,18,19], and governance in the implementation of the smart community [4,5,20,21,22,23]. Moreover, there are many kinds of studies on residents’ demands, most of which concentrate on a specific aspect, such as energy demand [24,25], elderly care services [26,27], medical care services [28,29], urban green space [30,31], public sports services [32], and housing demand [30,33]. However, relevant studies still suffer some limitations, which are embodied in the following three aspects. First, the demands for community services among residents in smart communities have failed to be classified systematically. Second, factors influencing residents’ expectations of community services in smart communities have rarely been examined. Third, a well-established theoretical framework has not yet been systematically applied to the analysis of residents’ needs and their determinants in smart communities.

Exploring the demands for various community services among residents in smart communities and their influencing factors is crucial to both enhance the efficiency of the service provision and overcome the deficiencies of the qualitative analyses performed previously. Hence, residents’ demands and characteristics have been taken into consideration in the service provision in this study, which seeks to: (1) classify various community services that residents need in smart communities; (2) explore the factors determining residents’ demands for a variety of community services; (3) identify ways for the improvement of the efficiency of community services.

This paper is further divided into the following sections: a review of literature is presented in Section 2 concerning residents’ needs for different types of community services in smart communities and relevant determinants, followed by 8 hypotheses and a comprehensive conceptual framework. The methods, questionnaire, and statistical models of this study are proposed in Section 3. Section 4 shows the basic information and demands of respondents, as well as the results of the binary logistic regression tests. Detailed explanations of the findings appear in Section 5. Section 6 summarizes the significance and policy implications of this study, along with a recommendation for future research.

## 2. Literature Review and Hypotheses

### 2.1. Different Types of Community Services in Smart Communities

People’s demands are structured according to their priorities based on Maslow’s Hierarchy of Needs (MHN). It has been used to account for the needs of hemodialysis patients and explore the demands of consumers for electric vehicles [34,35]. Derived from the MHN, the existence, relatedness, and growth (ERG) theory has been applied to analyze the demands of primary care workers in China [36,37]. Considering that residents’ demands for community services belong to human needs, the ERG theory is applicable to clarify the demands of residents in this study. On the basis of the MHN and the ERG theory, plenty of previous research related to community services has been referred to systematically classify residents’ demands. Community services in smart communities can be split into seven categories according to existing literature (i.e., smart business service [38,39,40,41,42,43], smart property service [38,39,40,44,45,46,47,48], smart emergency service [38,39,45,46,49,50], smart medical care service [38,45,51,52], smart elderly care service [38,39,53,54,55,56,57], smart communication service [39,40,45,58], and smart government service [40,44,58,59,60,61,62]), and there are a variety of services within each category [38,44,58]. A list of these services is provided in Table 1 (Detailed information about different types of community services can be seen in Supplementary File S1).

### 2.2. Factors Influencing Residents’ Demands for Community Services in Smart Communities

The attitude theory is a theory about the formation, transformation, and measure of people’s attitudes, which suggests that the attitude of people consists of cognition, affection, and behavioral intention [63,64]. It has been utilized to identify the determinants of the engagement of volunteers and clarify the impact of lack of mindfulness on impulse purchases during online shopping [63,65]. Considering that residents’ demands for community services in smart communities may be influenced by their attitudes, the influencing factors of residents’ demands were identified based on the attitude theory. Through a systematic review, the influencing factors related to residents’ demands for community services were identified as outlined in Table 2, which include sociodemographic characteristics (e.g., gender, age, career, educational level, marital status, and health status), living characteristics (e.g., living duration, living status, and housing choice), economic characteristics (e.g., monthly income and whether paying social insurance), and individual attitude characteristics (e.g., sense of gain, sense of safety, sense of happiness, perception of community services, and desire for smart community services) [66,67,68,69,70]. The detailed influencing factors are introduced in Supplementary File S2.

### 2.3. Hypotheses of this Study

Individuals’ sociodemographic characteristics have proved to be important factors in determining their needs according to previous studies. More specifically, men may require more medical care than women [26,67,70]. Seniors over the age of 70 are more likely to require meal assistance services [26,68,69]. There are distinct needs for on-call nursing and doctor services among residents from different professions [66]. Higher education may increase residents’ demands for medical care [26,66,67,68,69]. It is more likely for divorced people to seek medical care at hospitals than married people [68,69]. Those in poorer health may require more physical activities [26,66,69,70]. Hence, the first hypothesis is:

**Hypothesis** **1** **(H1).** *Residents’ demands for community services in smart communities will be significantly determined by their sociodemographic characteristics.*

Several studies indicate that residents’ living characteristics could affect their preferences. In more detail, people who reside in cities longer may be more inclined to stay there [67]. Healthcare services may be more popular with the elderly living with their spouses [26,69]. In comparison with house owners, renters prefer convenient public transportation [71]. Accordingly, the second hypothesis is the following:

**Hypothesis** **2** **(H2).** *Residents’ demands for community services in smart communities will be significantly determined by their living characteristics.*

There has been evidence in previous research that people’s economic characteristics influence their needs and choices. To be more specific, in terms of convenient medical care, residents with varying income levels may have different requirements [26,67,68,69,70]. Whether disabled seniors have medical insurance plays a significant role in their future choices of living [69,72]. Consequently, the third hypothesis is proposed:

**Hypothesis** **3** **(H3).** *Residents’ demands for community services in smart communities will be significantly determined by their economic characteristics.*

In accordance with the attitude theory, people’s cognition, affection, and behavioral intention may have distinct effects on their needs, which has been confirmed by the existing studies. Specifically, it has been discovered that learners’ satisfaction with their language learning is strongly influenced by their sense of gain [73]. The sense of safety of adolescents is significantly associated with their prosocial behaviors [74]. For entrepreneurs, the sense of happiness may play a role in their willingness to start a business [75]. Seniors’ perceptions of medical services significantly affect their demands [70]. The willingness of people to seek help is influenced by their desire for accessible services [76]. Thus, hypotheses 4 to 8 are made:

**Hypothesis** **4** **(H4).** *Residents’ demands for community services in smart communities will be significantly determined by their sense of gain.*

**Hypothesis** **5** **(H5).** *Residents’ demands for community services in smart communities will be significantly determined by their sense of safety.*

**Hypothesis** **6** **(H6).** *Residents’ demands for community services in smart communities will be significantly determined by their sense of happiness.*

**Hypothesis** **7** **(H7).** *Residents’ demands for community services in smart communities will be significantly determined by their perception of these services.*

**Hypothesis** **8** **(H8).** *Residents’ demands for community services in smart communities will be significantly determined by their desire for these services.*

**Figure 1:**
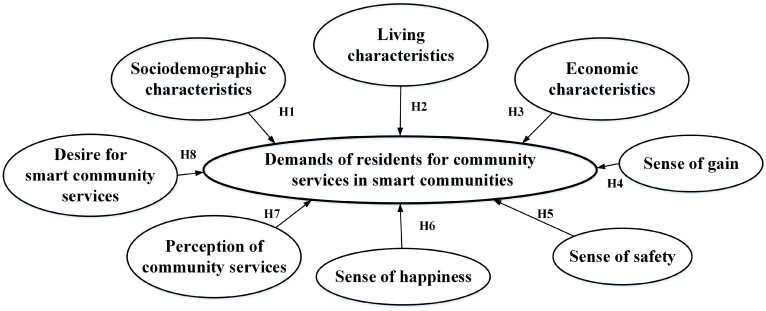
The conceptual framework for this research.

In light of these hypotheses, a comprehensive conceptual framework was developed to explore the factors that influence residents’ demands for a wide range of community services in smart communities, which is provided in Figure 1.

## 3. Method

The data of this research was collected by way of a questionnaire related to various variables and measures for the sake of quantitatively analyzing the differences among the needs of residents for community services in smart communities and identifying relevant determinants. The questionnaire is detailed in Supplementary File S3. Then, a quantitative survey was conducted online via the Wenjuanxing platform (https://www.wjx.cn/wjx/design/previewq.aspx?activity=162849986&s=1, accessed on 5 May 2022). Finally, the data analysis was completed by the chi-square (χ^2^) test and binary logistic regression.

### 3.1. Variables and Measures

Due to the lack of a standard validation survey concerning the demands of residents for community services in smart communities and the factors affecting them, a questionnaire to obtain the relevant information was designed.

Following are the sections of the final questionnaire:

1. A succinct explanation of community services in smart communities and the intention of this survey;
2. Respondents’ basic information;
3. The measurement of respondents’ demands for community services in smart communities. The question “Are you in need of this type of community service?” was used in the measurement and residents’ responses were measured as a dichotomous variable, with 1 representing a need for this type of community service and 0 otherwise. In light of the literature review, seven categories of community services in smart communities mentioned above were chosen as outcome variables and measured through residents’ responses to the question;
4. Factors influencing respondents’ demands for community services in smart communities, including respondents’ sociodemographic characteristics, living characteristics, economic characteristics, and individual attitude characteristics.

### 3.2. Sampling and Data Collection

Xuzhou city is one of the pilot cities for smart communities in eastern China which have been developed for nearly six years. Two reasons are taken into account in selecting Xuzhou as the study area. On the one hand, Xuzhou is one of the national pilot cities of community governance and innovative service provision [77]. In more detail, the Gulou District of Xuzhou has started to develop smart communities since late 2016, which has achieved fruitful results in terms of smart government services so far. For instance, a platform that provides various community services for residents has been developed and constantly upgraded by the government of Xuzhou, which has greatly facilitated residents’ daily lives [78,79]. Consequently, residents in Xuzhou may have a relatively better understanding of the community services identified above. On the other hand, as a result of the implementation of smart communities, residents in Xuzhou are becoming eager for community services in smart communities [80]. Thus, the differences in the demands of residents in Xuzhou can be readily detected during the data analysis.

Researchers have confirmed that the rule of 10 events per variable (EPV), which is a commonly used method for estimating minimum sample sizes, remains applicable for sampling despite acceptable levels of coverage and bias [81,82,83]. A minimum sample size of 160 was determined by applying the rule of 10 EPV according to the 16 variables clarified above. Considering the possibility of multiple deviations in this investigation, 240 questionnaires were sent to residents in Xuzhou online from May to June 2022. Data collected for this research were acquired from a survey administered through “Wenjuanxing”, which is an Internet platform that assists with the distribution of questionnaires to target respondents. Ultimately, 221 valid questionnaires were received in total, which exceeded the minimum sample size. Accordingly, the sample was sufficient to represent the overall residents in Xuzhou. 92.08% of the effective recovery was achieved.

### 3.3. Statistical Model and Analysis

It is necessary to conduct a difference analysis of respondents’ demands for various community services before exploring their influencing mechanisms, through which the identification of influencing factors that have different effects on respondents’ demands can be implemented. χ^2^ tests were performed to determine whether there were any deviations among the samples since dependent variables (categorical variables) were coded as 0 (no need) or 1 (need). Moreover, the statistical model was estimated by binary logistic regression, which is as follows:

(1) $$\ln\left(\frac{P_{j}}{1-P_{j}}\right)=\alpha +\overset{k}{\underset{i=1}{\sum }}\beta_{i}x_{i}+\overset{l}{\underset{i=1}{\sum }}\gamma_{i}y_{i}+\overset{m}{\underset{i=1}{\sum }}\lambda_{i}z_{i}+\overset{n}{\underset{i=1}{\sum }}\mu_{i}u_{i}+\varepsilon$$

In this model, j (j = 1, 2, …, 39) represents one of the community services in smart communities; Pj represents the probability of respondents’ demands for jth of community services; $\frac{Pj}{1-Pj}$ is the ‘odds ratio’; $\ln\frac{Pj}{1-Pj}$ is the log odds ratio, or ‘logit’; α is a random constant term; $x_{i}$ (*i* = 1, 2, …, 6) represents the sociodemographic characteristics of the respondents; $y_{i}$ (i = 1, 2, 3) represents the living characteristics of the respondents; $z_{i}$ (i = 1, 2) represents the economic characteristics of the respondents; $u_{i}$ (i = 1, 2, 3, 4, 5) represents the individual attitude characteristics of the respondents; $\beta_{i}$, $\gamma_{i}$, $\lambda_{i}$, and $\mu_{i}$ are coefficients corresponding to the independent variables mentioned above; the random error term is denoted by ε. Analyses of differences, regression, and data processing were carried out using SPSS 26.0 statistical software, which was created by Norman H. Nie et al in Palo Alto city of America. Moreover, the odds were computed to acquire odds ratios (OR).

## 4. Results

### 4.1. Descriptive Statistics of the Respondents

Respondents’ basic statistics are presented in Table 3. First of all, from the perspective of sociodemographic characteristics, the numbers of women and men were about equal with slightly more females than males (50.68%). Nearly half of the respondents were aged 18–35 (49.32%). As far as the occupation was concerned, the staff of state-owned enterprises and institutions accounted for the largest proportion (28.05%). Concerning the educational level, most of the respondents acquired a bachelor’s degree (57.92%). Additionally, married residents constituted the majority of the respondents (71.04%). In addition, the vast majority of the respondents were in good health (88.24%). Second, in terms of living characteristics, most respondents had lived in their community for more than 3 years (69.68%), did not live alone (93.67%), and were house owners (77.38%). Third, from the standpoint of economic characteristics, the respondents were divided according to their monthly incomes and 29.86% earned more than 7000 yuan (about 1026.92 USD) per month. Moreover, most respondents paid for all types of social insurance (66.52%). Fourth, taking into account individual attitude characteristics, most of the respondents agreed that their current lives in the community were full of senses of gain, security, and happiness (3.95, 4.09, and 3.97, respectively). As far as the degrees of perception of community services were concerned, community services were only partially understood by the majority of the respondents (3.37). The respondents who yearned for smart community services accounted for the largest proportion (4.31).

### 4.2. Residents’ Demands for Community Services in Smart Communities in Xuzhou

**Figure 2:**
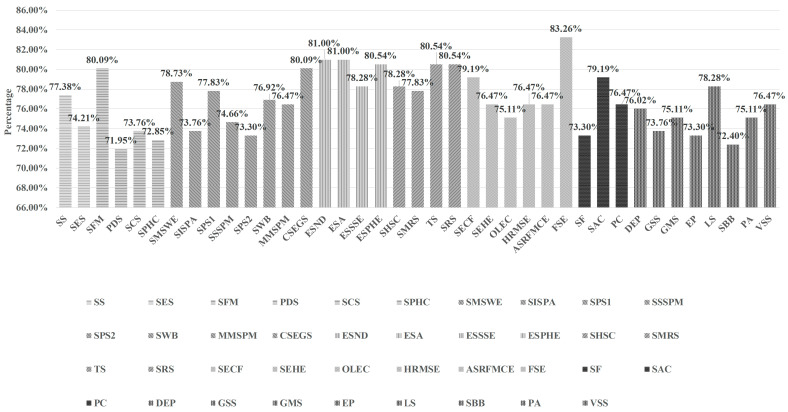
Respondents’ expressed demands for 39 types of community services in smart communities.

Respondents’ preferences for community services in smart communities are presented in Figure 2, which shows that almost all of the community services in smart communities were needed by more than 70% of the respondents and over 80% of the respondents needed the SFM (80.09%), the SEGS (80.09%), the ESND (81.00%), the ESA (81.00%), the ESPHE (80.54%), the TS (80.54%), the SRS (80.54%) and the FSE (83.26%).

Regarding the demands for smart business services, most respondents favored the SFM (80.09%) while the least favored the PDS (71.95%). The demands for the SS, the SES, the SCS, and the SPHC accounted for 77.38%, 74.21%, 73.76%, and 72.85%, respectively. In terms of smart property services, respondents had a higher demand for the SEGS (80.09%), while the demands for the SMSWE (78.73%), the SPS1 (77.83%), the SWB (76.92%), the MMSPM (76.47%), the SSSPM (74.66%), the SISPA (73.76%) and the SPS2 (73.30%) were reduced successively. What’s more, respondents indicated high levels of demand for smart emergency services. Specifically, the demands for the ESND and the ESA were the highest, both of which accounted for 81.00%, and the demand for the ESPHE also accounted for more than 80% (80.54%), while the demand for the ESSSE was the lowest (78.28%). Furthermore, smart medical care services were also in high demand among the respondents. Higher demands for the TS and the SRS were recorded, both of which exceeded 80% (80.54%), followed by the SHSC (78.28%) and the SMRS (77.83%). As far as the smart elderly care services were concerned, the most obvious was that respondents’ demand for the FSE accounted for as high as 83.26%, while others accounted for less than 80%. More specifically, the demands for the SECF (79.19%), the SEHE (76.47%), the HRMSE (76.47%), the ASRFMCE (76.47%), and the OLEC (75.11%) decreased successively. Concerning smart communication services, the demands of respondents varied greatly. In more detail, respondents’ demand for the SAC was the highest (79.19%), while the PC accounted for 76.47%, and the SF accounted for as low as 73.30%. Moreover, the demands of respondents for smart government services were generally low. Specifically, the LS with the highest demand proportion accounted for only 78.28%, while the SBB with the lowest demand proportion accounted for as low as 72.40%. The demands for the DEP (76.02%), the GSS (73.76%), the GMS (75.11%), the EP (73.30%), the PA (75.11%), and the VSS (76.47%) were reduced successively.

### 4.3. Results of the Binary Logistic Regression Test

According to experts, this study provides an in-depth analysis of community services in smart communities needed by more than 80% of the respondents. Hosmer and Lemeshow tests were conducted to assess the goodness of fit of these logistic regression models [84,85]. There was a sequential test for null hypothesis H0 (the model fits the data well) and alternative hypothesis H1 (it does not). If these models are well-fitted, Hosmer and Lemeshow’s test will return a *p*-value higher than 0.05. Analyses of the chi-square test and binary logistic regression of respondents’ demands for community services in smart communities needed by more than 80% of the respondents are shown in Table S3 in Supplementary File S4 and Table S4 in Supplementary File S5, respectively. According to Table S3, educational level (x14), marital status (x15), living duration (x21), housing choice (x23), monthly income (x31), sense of gain (x41), sense of safety (x42), perception of community services (x44) and desire for smart community services (x45) are nine factors that have significant differences in the demands of residents in Xuzhou for community services in smart communities (*p* < 0.05). Among these factors, educational level (x14) and marital status (x15) belong to sociodemographic characteristics (X1), living duration (x21) and housing choice (x23) belong to living characteristics (X2), and monthly income (x31) belongs to economic characteristics (X3). However, gender (x11), age (x12), career (x13), health status (x16), living status (x22), whether paying social insurance (x32), and sense of happiness (x43) of community residents in Xuzhou do not demonstrate significant differences in residents’ demands for community services in smart communities (*p* > 0.05). In summary, H1, H2, H3, H4, H5, H7, and H8 pass the preliminary difference test, while H6 fails the preliminary difference test.

#### 4.3.1. Assessment of Model Fit

As shown in Table S4, Hosmer and Lemeshow tests returned *p*-values greater than 0.05 for the remaining 8 models. Therefore, hypothesis H0 cannot be rejected for the SFM (y13), the SEGS (y28), the ESND (y31), the ESA (y32), the ESPHE (y34), the TS (y43), the SRS (y44) and the FSE (y56).

#### 4.3.2. Validation of Predicted Probabilities

**Figure 3:**
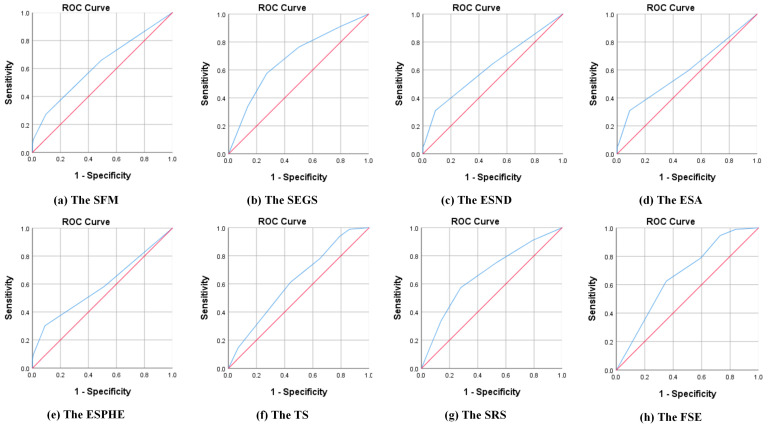
A combination of the ROC curves for initial demands and prediction performance. Note: The blue curves represent the ROC curves of the corresponding models, while the red curves represent the ROC curves of invalid models.

An illustration of whether the predicted probabilities match the actual results can be found in the classification table. Table S4 indicates that the odds of correct predictions for the y13, y28, y31, y32, y34, y43, y44, and y56 were 83.3, 81.9, 81.0, 81.9, 83.3, 81.0, 82.4, and 86.0% successively, which exceeded 80%. Clearly, predictions can be made with these 8 models. Moreover, the ROC curve provides a visualization of the accuracy of the prediction according to the relationship between the sensitivity and 1- specificity [86,87]. It is accepted that a larger area under the ROC curve translates into a better prediction for the model. The ROC curves of the 8 models that indicate respondents’ original demands and predicted probabilities are shown in Figure 3. From another perspective, the areas under the curves all exceeded 0.580, validating the good prediction performance of the models.

#### 4.3.3. Explanation of Coefficients in the Binary Logistic Regression

A total of 8 models related to the y13, y28, y31, y32, y34, y43, y44, and y56 were finalized after assessing the fits and validating the predicted probabilities of the models (Table S4). Table S4 indicates that the 8 primary demands were significantly (*p* < 0.05) associated with residents’ educational level (x14), monthly income (x31), perception of community services (x44), and desire for smart community services (x45).

In terms of the demand for the SFM, there was a strong correlation between this demand and the desire of respondents for smart community services (*p* < 0.01). Specifically, the probability of respondents having such a need was 0.432 times (OR = 0.432) lower for those with less desire for smart community services.

Respondents’ demand for the SEGS was significantly correlated with the monthly income (*p* < 0.05) and the desire for smart community services (*p* < 0.05). In more detail, respondents with higher monthly incomes showed an increased likelihood of having such demand by 1.486 times (OR = 1.486) according to the results. Furthermore, respondents with less desire for smart community services indicated a decreased likelihood of having such a need by 0.523 times (OR = 0.523).

As far as the demand for the ESND was concerned, our regression results demonstrated that it was significantly related to respondents’ perception of community services (*p* < 0.05) and desire for smart community services (*p* < 0.01). To be specific, those with less perception of community services revealed an increased likelihood of having such a need by 1.614 times (OR = 1.614). Additionally, there were 0.470 times (OR = 0.470) lower probability of respondents with less desire for smart community services having such a demand.

Taking into account the demand for the ESA, it is evident that this demand was influenced by the desire of respondents for smart community services (*p* < 0.01). More specifically, it was found that respondents with less desire for smart community services showed a decreased likelihood of having such a demand by 0.483 times (OR = 0.483).

As for the demand for the ESPHE, it was clear that respondents’ desire for smart community services had an impact on this demand (*p* < 0.05). In more detail, respondents with less desire for smart community services indicated a decreased likelihood of having such a need by 0.528 times (OR = 0.528).

With respect to the demand for the TS, there was no doubt that respondents’ educational level influenced this demand (*p* < 0.05). Specifically, respondents with higher educational levels were 1.451 times (OR = 1.451) more likely to have such a demand.

Taking a look at the demand for the SRS, significant impacts on this demand could be attributed to respondents’ educational level (*p* < 0.05) and monthly income (*p* < 0.05). To be specific, higher education level increased the likelihood of respondents having such a demand by 1.402 times (OR = 1.402). Moreover, respondents with higher monthly income revealed an increased likelihood of having such a need by 1.542 times (OR = 1.542).

From the perspective of the demand for the FSE, there was a significant impact of respondents’ educational level on this demand (*p* < 0.05). More specifically, respondents with higher education levels were 1.537 times (OR = 1.537) more likely to have such a demand.

To sum up, among the hypotheses that passed the preliminary difference test above, H1, H3, H7, and H8 passed the final binary logistic regression test, while H2, H4, and H5 failed the final binary logistic regression test.

## 5. Discussion

With the application of the χ^2^ test and binary logistic regression, the analysis of the survey data assisted with the quantification and comparison of residents’ demands for community services. Several key findings can be relied upon.

First, the demands of community residents in Xuzhou for smart government services were generally low, while the demands for survival community services were high, such as smart emergency services and smart medical services. It was found that the implementation of the smart community in Xuzhou was in-depth in terms of smart government services [78,79]. It is suggested in the theory of Maslow’s Hierarchy of Needs that people may reduce their demands if they were met. Therefore, one possible explanation for the low demands of residents for smart government services is that as the government has achieved fruitful results in the provision of smart government services, residents have reduced their demands for them, which is also in line with common sense [88]. Furthermore, the demands of residents for smart emergency services and smart medical services belong to the demand for existence that is mentioned in the ERG theory. Thus, one explanation for the high demands of residents for these two kinds of services is that residents may increase their demands for these two kinds of services to meet their need for existence [89]. Another explanation for residents’ high demand for these two kinds of services is that based on the attitude theory, residents may pay more attention to their health when they feel at risk of contracting the disease due to the pandemic of COVID-19 in recent years [90,91].

Second, one of the sociodemographic characteristics of residents, which is educational level, significantly affects their needs for community services in smart communities, considering the results of the binary logistic regression. In terms of the educational level, it significantly impacts respondents’ demands for the TS, the SRS, and the FSE. Specifically, these services, including basic medical care and elderly care services, are more likely to be requested by those with higher educational levels. There is a possibility that residents with a higher educational level are more concerned about their health status and eager for a comfortable elderly life [92].

Third, residents’ demands for community services in smart communities are significantly influenced by one of their economic characteristics, which is the monthly income, in accordance with the results of binary logistic regression analysis. With regard to the monthly income, there will be a significant impact on respondents’ demands for the SEGS and the SRS. In more detail, those with a higher monthly income have a higher demand for these community services, which encompass the improvement of living conditions and access to better medical care. One explanation for this phenomenon is that a higher monthly income may lead to residents’ higher requirements for their quality of life (including environmental quality and health quality) [93,94,95].

Fourth, some of the individual attitude characteristics of residents significantly influence their demands for community services in smart communities, such as their perception of community services and desire for smart community services according to the results of binary logistic regression analysis. Taking into account the perception of community services, it is likely that respondents’ demand for the ESND will be significantly affected by it. Specifically, residents with less perception of community services have a higher demand for the ESND. Regarding this phenomenon, one possible explanation according to the attitude theory is that respondents with less perception of community services may be more curious about these unfamiliar services [96,97]. As far as the desire for smart community services is concerned, respondents’ desire for smart community services will have a significant effect on their demands for the SFM, the SEGS, the ESND, the ESA, and the ESPHE. More specifically, residents who are more eager for smart community services have relatively higher demands for these community services, such as those related to the smart business service, smart property service, and smart emergency service. Considering this phenomenon, it can be interpreted based on the attitude theory that community services in smart communities could bring more intelligence and convenience to respondents’ daily lives; therefore, they were looking forward to their availability [98]. Nevertheless, it is worth noting that respondents’ desires for smart community services have no significant impact on their demands for smart medical care services, smart elderly care services, smart communication services, and smart government services. There is a possible explanation that the impacts of respondents’ desire for smart community services on their demands for these services are not linear. Therefore, the significance of these influencing mechanisms cannot be calculated by binary logistic regression [99,100].

## 6. Conclusions

Challenges occur in the transformation and upgrading of the community due to the continuous advancement of technology. This paper aims to systematically classify and give a better insight into residents’ needs for 39 types of community services in smart communities. A total of eight hypotheses were proposed and a conceptual framework was developed to explore and compare their demands and relevant determinants. Data from 221 residents in Xuzhou on the Internet were analyzed using the χ^2^ test and binary logistic regression. The statistical results from the survey indicated that almost all of the community services were needed by more than 70% of the respondents and over 80% of the respondents needed the SFM, the SEGS, the ESND, the ESA, the ESPHE, the TS, the SRS, and the FSE. Moreover, residents’ demands were affected by distinct factors, including their educational level, monthly income, perception of community services, and desire for smart community services. Therefore, H1, H3, H7, and H8 of the 8 hypotheses passed the verifications while the others failed. These findings assist with clarifying the differences in residents’ demands for community services in smart communities and promoting qualitative and descriptive analysis in the research of relevant determinants and influencing mechanisms. Additionally, the framework developed in this research can be tested in different setups depending on the differences among countries. Hence, the demands of residents in other countries for smart community services could be further explored according to the framework. Furthermore, reasonable policies could be implemented by the government to meet residents’ distinct demands and enhance the adaptability of service provision in different communities in line with this research.

For the purpose of developing a more popular smart community, several policy implications can be drawn from the above empirical findings. To begin with, smart communities need to improve their service delivery systems. Considering the lack of smart emergency services and smart medical care services in the service provision of Xuzhou and residents’ high demands for these two services, relevant services must be supplemented in accordance with the services classified in this study. Second, it is also important to pay attention to the individual needs of residents. It is essential for the government to build a smart community that meets the needs of residents according to the differences in their educational level, monthly income, perception of community services, and desire for smart community services of residents since residents play an important role in the community. Finally, publicity for community services in smart communities should be increased. In the future implementation of smart communities, relevant community services need to be publicized so that residents can have a deeper understanding of these services and their demands can be investigated with greater accuracy.

However, insignificant results need consideration as a result of the relatively small sample. In addition, more representative demands of residents failed to be collected due to the limitation of the research area. Moreover, field investigations were hampered by the pandemic of COVID-19, which may result in insufficient data collection and a reduction in the accuracy of the results. Larger-scale sets of data will be acquired in a similar study conducted in more cities in China in the future to achieve more reliable results.

## Figures and Tables

**Table 1:** A list of different types of community services in smart communities.

Category	Type	Researchers
Smart business service	1. Self-service Supermarket (SS)	[41,42]
	2. Smart E-commerce System (SES)	[38]
	3. Smart Farmers Market (SFM)	[43]
	4. Package Delivery System (PDS)	[38]
	5. Smart Childcare System (SCS)	[39]
	6. Service Platform for House Cleaning (SPHC)	[38,40]
Smart property service	1. Smart Management System of Water and Electricity (SMSWE)	[38,40]
	2. Smart Illumination System in Public Area (SISPA)	[38]
	3. Smart Parking System (SPS1)	[38,40]
	4. Smart Security System of Property Management (SSSPM)	[38,39,40,46]
	5. Smart Payment System (SPS2)	[40,44]
	6. Smart Waste Bin (SWB)	[47,48]
	7. Maintenance Management System of Property Management (CMMSPM)	[40]
	8. Smart Environmental Greening System (SEGS)	[39,40,45]
Smart emergency service	1. Emergency System of Natural Disaster (ESND)	[38,39,45,46]
	2. Emergency System of Accident (ESA)	[38,45,46]
	3. Emergency System of Social Security Event (ESSSE)	[49,50]
	4. Emergency System of Public Health Event (ESPHE)	[39]
Smart medical care service	1. Smart Healthcare Service Center (SHSC)	[45]
	2. Smart Medical Record System (SMRS)	[38]
	3. Telemedicine System (TS)	[38]
	4. Smart Referral System (SRS)	[51,52]
Smart elderly care service	1. Smart Elderly Care Facilities (SECF)	[38,53]
	2. Smart Elderly Health Examination (SEHE)	[38]
	3. Online Lectures about Elderly Care (OLEC)	[54,55]
	4. Health Record Management System of the Elderly (HRMSE)	[38]
	5. Appointment System of Regular and Free Medical Consultations for the Elderly (ASRFMCE)	[56,57]
	6. First-aid Service for the Elderly (FSE)	[39]
Smart communication service	1. Smart Forum (SF)	[39,40,58]
	2. Smart Activity Center (SAC)	[39,40,58]
	3. Psychological Counseling (PC)	[45]
Smart government service	1. Demand Expression Platform (DEP)	[40,58]
	2. Government Service System (GSS)	[44]
	3. Grid Management System (GMS)	[60]
	4. Employment Platform (EP)	[61]
	5. Legal Service (LS)	[62]
	6. Smart Bulletin Board (SBB)	[40,44]
	7. Poverty Assistance (PA)	[40]
	8. Volunteer Service System (VSS)	[59]

**Table 2:** A list of different types of factors influencing residents’ demands for community services in smart communities.

Category	Type	Researchers
Sociodemographic characteristics	1. Gender	[26,67,70]
	2. Age	[26,68,69]
	3. Career	[66]
	4. Educational level	[26,66,67,68,69]
	5. Marital status	[68,69]
	6. Health status	[26,66,69,70]
Living characteristics	1. Living duration	[67]
	2. Living status	[26,69]
	3. Housing choice	[71]
Economic characteristics	1. Monthly income	[26,67,68,69,70]
	2. Whether paying social insurance	[69,72]
Individual attitude characteristics	1. Sense of gain	[73]
	2. Sense of safety	[74]
	3. Sense of happiness	[75]
	4. Perception of community services	[70]
	5. Desire for smart community services	[76]

**Table 3:** Analyses of the sample using simple descriptive statistics.

Category	Type	Option	Frequency	Percentage (*N* = 221)
Sociodemographic characteristics	Gender	Male	109	49.32%
		Female	112	50.68%
	Age	17 years old and below	13	5.88%
		18–35 years old	109	49.32%
		36–45 years old	41	18.55%
		46–69 years old	58	26.24%
		70 years old and above	0	0.00%
	Career	Civil servant	14	6.33%
		Staff of state-owned enterprises and institutions	62	28.05%
		Staff of private and foreign enterprises and institutions	56	25.34%
		Individual industrial and commercial household	15	6.79%
		Freelancer	21	9.50%
		Student	41	18.55%
		Other	12	5.43%
	Educational level	Primary school or below	8	3.62%
		Middle school	12	5.43%
		High school and technical secondary school	34	15.38%
		Junior college	39	17.65%
		Bachelor’s degree	99	44.80%
		Master’s degree or above	29	13.12%
	Marital status	Married	157	71.04%
		Unmarried	64	28.96%
	Health status	Good	195	88.24%
		General	25	11.31%
		Bad	1	0.45%
Living characteristics	Living duration	Less than 1 year	23	10.41%
		1 to 3 years	44	19.91%
		More than 3 years	154	69.68%
	Living status	Living alone	14	6.33%
		Not living alone	207	93.67%
	Housing choice	Renter	28	12.67%
		House owner	171	77.38%
		Other	22	9.95%
Economic characteristics	Monthly income	Within 1000 RMB (about 146.69 USD)	25	11.31%
		1000–3000 RMB (about 146.69–440.09 USD)	39	17.65%
		3000–5000 RMB (about 440.09–733.54 USD)	43	19.46%
		5000–7000 RMB (about 733.54–1026.92 USD)	48	21.72%
		Above 7000 RMB (about 1026.92 USD)	66	29.86%
	Whether paying social insurance	All	147	66.52%
		Partly (e.g., only medical insurance)	50	22.62%
		Not at all	24	10.86%
Individual attitude characteristics	Sense of gain	Mean score of sense of gain	3.95	
	Sense of safety	Mean score of sense of safety	4.09	
	Sense of happiness	Mean score of sense of happiness	3.97	
	Perception of community services	Mean score of perception of community services	3.37	
	Desire for smart community services	Mean score of desire for smart community services	4.31	

## Data Availability

Please contact the corresponding authors for access to the data presented in this study. Due to the protection of respondents’ privacy, the data are not publicly available.

## Supplementary Materials

The following supporting information can be downloaded at https://www.mdpi.com/article/10.3390/ijerph20043750/s1: Table S1: A list of different types of community services in smart communities; Table S2: A list of different types of factors influencing residents’ demands for community services in smart communities; File S3: Questionnaire on demands of residents for community services in smart communities. Table S3: Chi square analysis results of demands for community services in smart communities with a demand proportion of more than 80%; Table S4: Binary logistic analysis results of demands for community services in smart communities with a demand proportion of more than 80%.

## Abbreviations

SS	Self-service Supermarket
SES	Smart E-commerce System
SFM	Smart Farmers Market
PDS	Package Delivery System
SCS	Smart Childcare System
SPHC	Service Platform for House Cleaning
SMSWE	Smart Management System of Water and Electricity
SISPA	Smart Illumination System in Public Area
SPS1	Smart Parking System
SSSPM	Smart Security System of Property Management
SPS2	Smart Payment System
SWB	Smart Waste Bin
MMSPM	Maintenance Management System of Property Management
CSEGS	Smart Environmental Greening System
ESND	Emergency System of Natural Disaster
ESA	Emergency System of Accident
ESSSE	Emergency System of Social Security Event
ESPHE	Emergency System of Public Health Event
SHSC	Smart Healthcare Service Center
SMRS	Smart Medical Record System
TS	Telemedicine System
SRS	Smart Referral System
SECF	Smart Elderly Care Facilities
SEHE	Smart Elderly Health Examination
OLEC	Online Lectures about Elderly Care
HRMSE	Health Record Management System of the Elderly
ASRFMCE	Appointment System of Regular and Free Medical Consultations for the Elderly
FSE	First-aid Service for the Elderly
SF	Smart Forum
SAC	Smart Activity Center
PC	Psychological Counseling
DEP	Demand Expression Platform
GSS	Government Service System
GMS	Grid Management System
EP	Employment Platform
LS	Legal Service
SBB	Smart Bulletin Board
PA	Poverty Assistance
VSS	Volunteer Service System
