# Supplementary material for: Demands for Community Services and Associated Factors among Residents in Smart Communities: A Case Study of Xuzhou City
Source: Int J Environ Res Public Health. 2023 Feb 20;20(4):3750. doi: 10.3390/ijerph20043750 (PMC9964050; doi:10.3390/ijerph20043750)
Supplement: Supplementary file 1 [file ijerph-20-03750-s001.zip › Supplementary File S1.pdf]

Table S1. A list of different types of community services in smart communities.

Category	Type	Brief description	Researchers
Smart business service	1. Self-service Supermarket (SS)	A community supermarket with the functions of facial access control, self-service selection of goods, and self-service settlement, which can greatly save human resources, improve settlement efficiency, improve the efficiency and speed of residents' shopping, and save residents' shopping time.	[1,2]
	2. Smart E-commerce System (SES)	An O2O e-commerce system that integrates the businesses of clothing, food, housing, and transportation in the community to facilitate the development of community business.	[3]
	3. Smart Farmers Market (SFM)	A community farmer's market which has a variety of functions such as intelligent gates, intelligent collection, intelligent detection, intelligent weighing, intelligent cashier, vegetable price transaction announcement, and merchant information announcement. It can improve operational efficiency, save the shopping time of customers, and save human resources.	[4]
	4. Package Delivery System (PDS)	A package delivery system that uses mobile terminals and intelligent express cabinets as platforms. It provides residents in the community with information about package collection and delivery. Besides, there are a variety of functions of the system such as data storage, intelligent monitoring, face recognition, and online payment, which can save time for residents to receive and send packages to improve the efficiency of package delivery.	[3]
	5. Smart Childcare System (SCS)	A kindergarten system with mobile terminals, teacher watches, and student bracelets. It can provide infant care and school-age child care services, which has a variety of functions such as face recognition, behavior records,	[5]

Category	Type	Brief description	Researchers
		emergency calls, intelligent monitoring, and home interaction to reduce the burden on parents in the community.	
	6. Service Platform for House Cleaning (SPHC)	A service platform where residents can book cleaning services, choose the type of service, and pay online. The platform can record service information, make the service process traceable, and link community businesses to create economic growth and bring convenience to residents.	[3,6]
Smart property service	1. Smart Management System of Water and Electricity (SMSWE)	A household water and electricity management system in the community composed of smart meters and remote meter reading software, which has a variety of functions such as record management, intelligent switching, data statistics, reminder service, and intelligent alarm. It is of high reliability and stable performance and can save human resources and improve the supply and management efficiency of water and electricity.	[3,6]
	2. Smart Illumination System in Public Area (SISPA)	A community illumination system composed of the terminal layer, network layer, platform layer, and application layer, which can make community illumination more intelligent and user-friendly through the use of the light sensor, dimming module, and the link of information collection, personnel positioning, traffic control, etc.	[3]
	3. Smart Parking System (SPS1)	A community parking lot with advanced functions such as automatic license plate recognition, video parking guidance, unattended management, and intelligent monitoring that can improve the efficiency of the traffic, and can enrich the ways of payment in the case of linkage with the intelligent payment system. It is convenient for parking space positioning and vehicle positioning, which can reduce operating costs and save human resources.	[3,6]
	4. Smart Security System of	A community security system composed of a video surveillance system,	[5-8]

Category	Type	Brief description	Researchers
	Property Management (SSSPM)	access control system, face recognition system, intrusion alarm system, wireless intercom system, and broadcasting system, which can dynamically monitor the security situation of the community.	
	5. Smart Payment System (SPS2)	A payment system using mobile terminals and websites, which has a variety of functions such as online payment, online automatic reminder, and intelligent management. In this system, residents can pay their daily expenses, such as gas, parking, and property management, which can save residents' time, save human resources, and improve the efficiency of payment.	[6,9]
	6. Smart Waste Bin (SWB)	A community waste bin with a variety of functions such as full bucket prompt, classified storage, garbage weighing, automatic door opening, voice prompt, monitoring system, smoke alarm, fire extinguishing device, automatic networking, and solar charging. It is convenient for community sanitation workers to dispose of garbage, which helps to improve the community environment and save human resources.	[10,11]
	7. Maintenance Management System of Property Management (MMSPM)	A maintenance management system using mobile terminals and websites, where the owner can track the repair progress in real-time and evaluate the repair service in the system. It can improve the motivation and maintenance efficiency of the workers while optimizing the allocation of human resources.	[6]
	8. Smart Environmental Greening System (SEGS)	An environmental greening system with the functions of air quality detection, temperature monitoring, automatic spray cooling, intelligent voice prompts, and intelligent water-saving sprinkler irrigation, which can improve the community environment, improve the air quality of the	[5,6,12]

Category	Type	Brief description	Researchers
		community, and facilitate the management of sanitation workers.	
Smart emergency service	1. Emergency System of Natural Disaster (ESND)	A community emergency system composed of fire emergency lighting, evacuation instructions, flood alarm, drought alarm, earthquake alarm, weather warning, and centralized control system, which has the functions of intelligent monitoring, data recording, analysis of escape paths, broadcast evacuation, emergency lighting, and intelligent instructions. The system can detect safety problems in time and ensure the personal and property safety of the community residents. It is used to deal with natural disasters in the community.	[5,7,8,12]
	2. Emergency System of Accident (ESA)	A community emergency system composed of fire emergency lighting, evacuation instructions, fire alarm, traffic accident alarm, ecological damage alarm, and centralized control system, which has the functions of intelligent monitoring, data recording, analysis of escape paths, broadcast evacuation, emergency lighting, and intelligent instructions. The system can detect safety problems in time and ensure the personal and property safety of the community residents. It is used to deal with accidents in the community.	[7,8,12]
	3. Emergency System of Social Security Event (ESSSE)	A community emergency system with functions such as population statistics, fraud warning, crime handling, traffic management, security management, emergency alarm button, and mobile police response to ensure the personal and property safety of the community residents. It is used to deal with social security events in the community.	[13,14]
	4. Emergency System of Public Health Event (ESPHE)	A community emergency system which provides first-aid services to deal with epidemics, sudden diseases of residents, and other public health events that seriously affect the health and safety of residents. It links community	[5]

Category	Type	Brief description	Researchers
		hospitals and 120 first-aid centers, uses computer-aided decision-making to formulate and implement first-aid plans, and improves the efficiency of first-aid work.	
Smart medical care service	1. Smart Healthcare Service Center (SHSC)	A healthcare service center which has smart medical functions (diagnosis and treatment appointments, health examinations, vaccinations, intelligent payment, intelligent triage, mobile phone registration, laboratory test interpretation, artificial intelligence-assisted diagnosis, clinic department navigation, etc.), smart nursing functions (intelligent infusion, intelligent bed, temperature monitoring, voice recognition, face recognition, etc.), and smart management functions (information storage, intelligent retrieval, etc.) that can improve the efficiency of diagnosis, treatment, and management.	[12]
	2. Smart Medical Record System (SMRS)	A health record system for community residents using mobile terminals and websites. Patients can view their appointment records, medical treatment records, medication history, related expenses, online consultation records, and other information through mobile terminals in this system. Community residents can not only check their health status in time but also consult with 24-hour online doctors.	[3]
	3. Telemedicine System (TS)	A telemedicine system using mobile terminals and websites, which has a variety of functions such as video visits, remote consultations, and e-prescriptions. It can avoid direct contact between doctors and patients, prevent the spread of infectious diseases, support the allocation of community medical resources, and help patients receive medical treatment in time.	[3]
	4. Smart Referral System	A referral system that includes referral services such as association	[15,16]

Category	Type	Brief description	Researchers
	(SRS)	management, referral management, and data statistics, which can quickly connect different hospitals, optimize resource allocation, and help patients receive medical treatment in time.	
Smart elderly care service	1. Smart Elderly Care Facilities (SECF)	A nursing service system for the elderly in the community using mobile terminals, which has a variety of functions such as vital sign collection, infusion monitoring, intelligent bracelets, face recognition, health management, and dietary management. It can help to save human resources and improve the efficiency and quality of community elderly care services.	[7,17]
	2. Smart Elderly Health Examination (SEHE)	A health examination system for the elderly in the community using mobile terminals, which is composed of an intelligent health examination guide system and a health management system that can collect the health information of the elderly in time.	[3]
	3. Online Lectures about Elderly Care (OLEC)	Online lectures about elderly care and health held through mobile terminals and websites. Experts from community hospitals are invited to give speeches. Community elderly residents can watch the lectures online within the specified time. Hospital experts can popularize elderly care and health knowledge on the platform, which can save human resources and improve the efficiency of publicity.	[18,19]
	4. Health Record Management System of the Elderly (HRMSE)	A record management system established by elderly care institutions and hospitals for the elderly in the community, which has functions such as health information recording, face recognition, and health status monitoring. It can store the health information of the elderly in the community and facilitate nursing work.	[3]
	5. Appointment System of	A free medical consultation appointment system established by elderly care	[20,21]

Category	Type	Brief description	Researchers
	Regular and Free Medical Consultations for the Elderly (ASRFMCE)	institutions and hospitals for the elderly in the community, which uses mobile terminals. Community elderly care institutions and hospitals can organize free medical consultation activities and publish relevant information on the platform. The elderly can make an appointment in the system and go to offline designated locations to receive medical services on the specified date or receive medical services remotely online directly, which is convenient for the elderly residents to treat their daily ailments.	
	6. First-aid Service for the Elderly (FSE)	A kind of first-aid service provided by elderly care institutions and hospitals for the elderly in the community, which uses mobile terminals like intelligent bracelets. It has a variety of functions such as intelligent health monitoring, "one button call" and GPS positioning that can help to take care of the life and health of the elderly residents in the community.	[5]
Smart communication service	1. Smart Forum (SF)	A communication forum provided by the community for residents, which uses mobile terminals and community websites. Residents can discuss community problems, seek solutions, put forward relevant suggestions, make friends, and browse community news on this forum. Community officials can publish community news, answer residents' questions and collect residents' opinions and suggestions on this forum, which can improve the efficiency of community governance.	[5,6,22]
	2. Smart Activity Center (SAC)	An activity center provided by the community for residents with the functions of a wireless network, intelligent guide, vending machine, intelligent broadcasting, children's space, painting wall, book corner, intelligent study space, seat reservation, face recognition, intelligent drinking fountains, intelligent temperature control, and intelligent	[5,6,22]

Category	Type	Brief description	Researchers
		illumination systems. With the characteristics of digital operation and intelligent management, it can create a good communication, reading and learning environment for the community residents.	
	3. Psychological Counseling (PC)	A platform providing psychological counseling services for residents in the community using mobile terminals and websites. Residents can consult about psychological problems and seek psychological assistance on this platform. The community can set up a psychological hotline on the platform and volunteers can provide voluntary psychological services for residents on this platform.	[12]
Smart government service	1. Demand Expression Platform (DEP)	A platform provided by the community for residents to express their demands to the government, which uses mobile terminals and websites. Two-way communication between the government and residents can be realized on this platform. It can broaden the channels for the government to communicate with the public and improve the efficiency of the provision of government services.	[6,22]
	2. Government Service System (GSS)	A system where the community provides government services to residents using mobile terminals or websites. Residents can inquire about government policies and handle related businesses on this platform. The community government can publish the latest policies, announcements, and other information in the system.	[23]
	3. Grid Management System (GMS)	A community management system that divides the community into several grids and connects to various business systems to promote collaborative work among various units. The system associates residents with their living places. Grid managers can upload affairs that occur in the community	[24]

Category	Type	Brief description	Researchers
		through mobile terminals and assign them to relevant service departments or organizations for solutions.	
	4. Employment Platform (EP)	A platform publishing employment information provided by the community for residents using mobile terminals and websites. Residents can search for employment opportunities on this platform, and employers can publish their recruitment information on it at the same time.	[25]
	5. Legal Service (LS)	A platform providing legal consulting services by the community for residents using mobile terminals and websites. Residents can consult legal issues and look for legal assistance on the platform. Law firms or related non-profit organizations in the community can also provide paid or voluntary legal services to residents on it.	[26]
	6. Smart Bulletin Board (SBB)	An intelligent electronic bulletin board placed in the community which has the functions of live TV, voice broadcast, and searching for surrounding business districts that can display commercial advertisements, public service advertisements, and government propaganda films.	[6,27]
	7. Poverty Assistance (PA)	Services provided by the community for people with financial difficulties, disabled people, relatives of martyrs, and other residents in need. A platform is built using mobile terminals and websites to collect information about these residents. In addition, daily supplies and living allowances are regularly distributed to them.	[6]

Category	Type	Brief description	Researchers
	8. Volunteer Service System (VSS)	A volunteer service system provided by the community for residents using mobile terminals and websites, which has the functions of volunteer recruitment, information recording, and volunteer time statistics. It is sponsored by commercial companies and led by the government in the community. Volunteer activity publishers can publish volunteer activities in the system, while volunteers can participate in activities, accumulate volunteer points, and enjoy the welfare provided by the community.	[28]

References:

Mo, L.; Li, C. Passive Uhf-Rfid Localization Based On the Similarity Measurement of Virtual Reference Tags. *Ieee Trans. Instrum. Meas.* **2019**, 68, 2926-2933.

Sattlegger, L. Making Food Manageable - Packaging as a Code of Practice for Work Practices at the Supermarket. *J. Contemp. Ethnogr.* **2021**, 50, 341-367.

Qi, L.; Guo, J. Development of Smart City Community Service Integrated Management Platform. *Int. J. Distrib. Sens. Netw.* **2019**, 15, 370582068.

Nuss, H.; Skizim, M.; Afaneh, H.; Miele, L.; Sothern, M. Farmers' Market Utilization Among Supplemental Nutrition Assistance Program Recipients in New Orleans, Louisiana: Preliminary Findings. *Ethn. Dis.* **2017**, 271, 295-302.

Li, X.; Lu, R.; Liang, X.; Shen, X.S.; Chen, J.; Lin, X. Smart Community: An Internet of Things Application. *Ieee Commun. Mag.* **2011**, 49, 68-75.

Mital, M.; Pani, A.K.; Damodaran, S.; Ramesh, R. Cloud Based Management and Control System for Smart Communities: A Practical Case Study. *Comput. Ind.* **2015**, 74, 162-172.

Qi, L.; Guo, J. Development of Smart City Community Service Integrated Management Platform. *Int. J. Distrib. Sens. Netw.* **2019**, 15, 370582068.

Jiang, H. Design and Implementation of Smart Community Big Data Dynamic Analysis Model Based On Logistic Regression Model. *Comput. Intell. Neurosci.* **2022**, 2022, 4038084.

Li, R.; Huang, Q.; Chen, X.; Zheng, B.; Liu, H. Factors Affecting Smart Community Service Adoption Intention: Affective Community Commitment and Motivation Theory. *Behav. Inf. Technol.* **2019**, 38, 1324-1336.

Pardini, K.; Rodrigues, J.J.P.C.; Diallo, O.; Das, A.K.; de Albuquerque, V.H.C.; Kozlov, S.A. A Smart Waste Management Solution Geared Towards Citizens. *Sensors* **2020**, 20, 23808.

Zhang, Q.; Li, H.; Wan, X.; Skitmore, M.; Sun, H. An Intelligent Waste Removal System for Smarter Communities. *Sustainability* **2020**, 12, 682917.

Wang, F.; Zhang, J.; Zhang, P. Influencing Factors of Smart Community Service Quality: Evidence From China. *Teh. Vjesn.* **2021**, 28, 1187-1196.

Corral-De-Witt, D.; Carrera, E.V.; Munoz-Romero, S.; Luis Rojo-Alvarez, J. Statistical, Spatial and Temporal Mapping of 911 Emergencies in Ecuador. *Appl. Sci.-Basel* **2018**, 8, 1992.

Ansari, S.; Yoon, S.; Albert, L.A. An Approximate Hypercube Model for Public Service Systems with Co-Located Servers and Multiple Response. *Transp. Res. Pt. E-Logist. Transp. Rev.* **2017**, 103, 143-157.

Buitron De La Vega, P.; Losi, S.; Martinez, L.S.; Bovell-Ammon, A.; Garg, A.; James, T.; Ewen, A.M.; Stack, M.; DeCarvalho, H.; Sandel, M.,

et al. Implementing an Ehr-Based Screening and Referral System to Address Social Determinants of Health in Primary Care. *Med. Care* **2019**, 572, S133-S139.

Doshmangir, L.; Bazyar, M.; Majdzadeh, R.; Takian, A. So Near, so Far: Four Decades of Health Policy Reforms in Iran, Achievements and Challenges. *Arch. Iran. Med.* **2019**, 22, 592-605.

Alabdulatif, A.; Khalil, I.; Forkan, A.R.M.; Atiquzzaman, M. Real-Time Secure Health Surveillance for Smarter Health Communities. *Ieee Commun. Mag.* **2019**, 57, 122-129.

Sahi, P.K.; Mishra, D.; Singh, T. Medical Education Amid the Covid-19 Pandemic. *Indian Pediatrics* **2020**, 57, 652-657.

Camargo, C.P.; Tempski, P.Z.; Busnardo, F.F.; Martins, M.D.A.; Gemperli, R. Online Learning and Covid-19: A Meta-Synthesis Analysis. *Clinics* **2020**, 75, e2286.

Iyal, H.A.; Shuaib, F.; Dauda, M.; Suleiman, A.; Braka, F.; Tegegne, S.G.; Nsubuga, P.; Nomhwange, T.; Yehualashet, Y.G.; Ishaku, S., et al. Assessment of Unmet Needs to Address Noncompliant Households During Polio Supplemental Immunization Activities in Kaduna State, 2014-2016. *Bmc Public Health* **2018**, 184, 1309.

Higuchi, M.; Endo, M.; Yoshino, A. Factors Associated with Access to Health Care Among Foreign Residents Living in Aichi Prefecture, Japan: Secondary Data Analysis. *Int. J. Equity Health* **2021**, 20, 1351.

Zhang, Y.; Zhao, H.; Hu, Y.; Yao, G. Smart Community Service Brand Functional Value and Sustainable Brand Relationship-the Mediating Role of Customer Emotional Cognition. *Sustainability* **2021**, 13, 18334.

Li, R.; Huang, Q.; Chen, X.; Zheng, B.; Liu, H. Factors Affecting Smart Community Service Adoption Intention: Affective Community

Commitment and Motivation Theory. *Behav. Inf. Technol.* **2019**, 38, 1324-1336.

Mittelstaedt, J.C. The Grid Management System in Contemporary China: Grass-Roots Governance in Social Surveillance and Service Provision. *China Inf.* **2022**, 36, 3-22.

Wheeler, L.; Garlick, R.; Johnson, E.; Shaw, P.; Gargano, M. Linkedin(to) Job Opportunities: Experimental Evidence From Job Readiness Training. *Am. Econ. J.-Appl. Econ.* **2022**, 14, 101-125.

Alarie, B.; Niblett, A.; Yoon, A.H. How Artificial Intelligence Will Affect the Practice of Law. *Univ. Toronto Law J.* **2018**, 681, 106-124.

Li, R.; Huang, Q.; Chen, X.; Zheng, B.; Liu, H. Factors Affecting Smart Community Service Adoption Intention: Affective Community Commitment and Motivation Theory. *Behav. Inf. Technol.* **2019**, 38, 1324-1336.

Guo, J.; Ling, W. Impact of Smart City Planning and Construction On Community Governance Under Dynamic Game. *Complexity* **2021**, 2021, 6690648.
