# Supplementary material for: Demands for Community Services and Associated Factors among Residents in Smart Communities: A Case Study of Xuzhou City
Source: Int J Environ Res Public Health. 2023 Feb 20;20(4):3750. doi: 10.3390/ijerph20043750 (PMC9964050; doi:10.3390/ijerph20043750)
Supplement: Supplementary file 1 [file ijerph-20-03750-s001.zip › Supplementary File S2.pdf]

Table S2. A list of different types of factors influencing residents' demands for community services in smart communities.

Category	Type	Brief description	Researchers
Sociodemographic characteristics	1. Gender	The gender of community residents may have an impact on their demands for community services in smart communities. In more detail, men may have higher demands for medical services.	[1-3]
	2. Age	The age of community residents may have an impact on their demands. In more detail, the demands for meal-aid services are higher among the elderly over the age of 70.	[1,4,5]
	3. Career	The career of community residents may have an impact on their demands for community services in smart communities. In more detail, the residents, who worked in the field of agriculture, forestry, animal husbandry, and fishing, as well as those who worked in public institutions or who worked in state-owned companies and private companies before they retired, might have lower demands for on-call nursing and doctor visits.	[6]
	4. Educational level	The educational level of community residents may have an impact on their demands for community services in smart communities. In more detail, the elderly with more education may have higher demands for on-call nursing and doctor visits.	[1,2,4-6]
	5. Marital status	The marital status of community residents may have an impact on their demands for community services in smart communities. In more detail, compared with married couples, a divorced elderly person is more willing to go to the general hospital.	[4,5]
	6. Health status	The health status of community residents may have an impact on their demands for community services in smart communities. In more detail, the elderly with poorer health may have a higher demand for sporting fitness.	[1,3,5,6]
Living characteristics	1. Living	The living duration of community residents may have an impact on their demands	[2]

Category	Type	Brief description	Researchers
	duration	for community services in smart communities. In more detail, migrants who have lived in cities longer may be more willing to stay in cities.	
	2. Living status	The living status of community residents may have an impact on their demands for community services in smart communities. In more detail, the elderly living with their spouses may have higher demands for the elderly care hotline and exercise.	[1,5]
	3. Housing choice	The housing choice of community residents may have an impact on their demands for community services in smart communities. In more detail, renters have a higher demand for convenient public transportation than house owners.	[7]
Economic characteristics	1. Monthly income	The monthly income of community residents may have an impact on their demands for community services in smart communities. In more detail, residents of different income levels may have different demands for family doctor services.	[1-5]
	2. Whether paying social insurance	The choice of community residents whether to pay social insurance or not may have an impact on their demands for community services in smart communities. In more detail, the future living arrangements of the disabled elderly are closely related to whether they pay for medical insurance.	[5,8]
Individual attitude characteristics	1. Sense of gain	The sense of gain of community residents may have an impact on their demands for community services in smart communities. In more detail, the sense of gain is found to exert a significant direct effect on learners' language learning enjoyment who takes Chinese as a second language.	[9]
	2. Sense of safety	The sense of safety of community residents may have an impact on their demands for community services in smart communities. In more detail, there is a significant correlation between the sense of safety and the prosocial behavior of adolescents.	[10]
	3. Sense of happiness	The sense of happiness of community residents may have an impact on their demands for community services in smart communities. In more detail, the sense of	[11]

Category	Type	Brief description	Researchers
		happiness may influence the willingness of female entrepreneurs to start businesses.	
	4. Perception of community services	The perception of community services of community residents may have an impact on their demands for community services in smart communities. In more detail, the perception of community medical services significantly affects the demands of community elderly people for community medical services.	[3]
	5. Desire for smart community services	The desire of community residents for smart community services may have an impact on their demands for community services in smart communities. In more detail, the willingness of intimate partner violence survivors to seek help is influenced by their desire for accessible services.	[12]

References:

Gu, T.; Li, D.; Li, L. The Elderly's Demand for Community-Based Care Services and its Determinants: A Comparison of the Elderly in the Affordable Housing Community and Commercial Housing Community of China. *J. Healthc. Eng.* **2020**, 2020, 1840543.

Liu, T.; Xiao, W. The 'Intention to Stay' of the Floating Migrant Population: A Spatiotemporal Meta-Analysis in Chinese Cities. *Popul. Space Place.* **2022**, 28, e2574.

Yu, Z.; Wang, L.; Ariyo, T. Supply and Demand-Related Decisive Factors in the Utilization of Non-Medical Community Healthcare Services Among Elderly Chinese. *Int. J. Environ. Res. Public Health* **2021**, 18, 2281.

Shang, X.; Huang, Y.; Li, B.; Yang, Q.; Zhao, Y.; Wang, W.; Liu, Y.; Lin, J.; Hu, C.; Qiu, Y. Residents' Awareness of Family Doctor Contract Services, Status of Contract with a Family Doctor, and Contract Service Needs in Zhejiang Province, China: A Cross-Sectional Study. *Int. J.*

Environ. Res. Public Health **2019**, 16, 331218.

Yang, S.; Wang, D.; Li, C.; Wang, C.; Wang, M. Medical Treatment Behaviour of the Elderly Population in Shanghai: Group Features and Influencing Factor Analysis. *Int. J. Environ. Res. Public Health* **2021**, 18, 41088.

Gu, T.; Yuan, J.; Li, L.; Shao, Q.; Zheng, C. Demand for Community-Based Care Services and its Influencing Factors Among the Elderly in Affordable Housing Communities: A Case Study in Nanjing City. *Bmc Health Serv. Res.* **2020**, 20, 2411.

Cui, N.; Gu, H.; Shen, T.; Feng, C. The Impact of Micro-Level Influencing Factors On Home Value: A Housing Price-Rent Comparison. *Sustainability* **2018**, 10, 434312.

Zhang, J.; Wang, Z.; Lin, L.; Huang, J.; Dong, Y.; Li, H. Determinants of Preference for Future Living Arrangement in Disabled Elders: A Cross-Sectional Study of Elderly Residents in Kunshan, China. *Psychogeriatrics* **2022**, 22, 324-331.

Zhang, L.; Tsung, L. Learning Chinese as a Second Language in China: Positive Emotions and Enjoyment. *System* **2021**, 96, 102410.

Seidler, C.; Rau, T.; Allroggen, M. The Relationship Between a Sense of Security and Psychological Problems in Adolescents Living in Boarding Schools and Youth-Welfare Institutions. *Z. Kinder-Und Jugendpsy. Psychother.* **2018**, 46, 230-237.

De Clercq, D.; Kaciak, E.; Thongpapanl, N. Happy at Home, Successful in Competition: The Beneficial Role of Happiness and Entrepreneurial Orientation for Women Entrepreneurs. *Int. J. Entrep. Behav. Res.* **2022**, 28, 1463-1488.

Ravi, K.E.; Robinson, S.R.; Schrag, R.V. Facilitators of Formal Help-Seeking for Adult Survivors of Ipv in the United States: A Systematic Review. *Trauma Violence Abus.* **2021**, 23, 1420-1436.
