# Supplementary material for: Demands for Community Services and Associated Factors among Residents in Smart Communities: A Case Study of Xuzhou City
Source: Int J Environ Res Public Health. 2023 Feb 20;20(4):3750. doi: 10.3390/ijerph20043750 (PMC9964050; doi:10.3390/ijerph20043750)
Supplement: Supplementary file 1 [file Supplementary File S4.pdf › Supplementary File S4.pdf]

Table S3. Chi square analysis results of demands for community services in smart communities with a demand proportion of more than 80%.

Category	Type	SFM	SEGS	ESND	ESA	ESPHE	TS	SRS	FSE
Sociodemographic characteristics	Gender	2.509	1.555	1.623	1.623	3.133	0.169	2.046	0.398
	Age	1.524	2.240	2.767	3.146	4.701	5.407	4.547	5.431
	Career	3..718	9.765	7.971	5.768	3.733	2.293	4.060	0.954
	Educational level	9.920	10.446	14.897*	11.756*	12.576*	18.604**	15.098**	21.107***
	Marital status	0.218	1.464	4.868*	4.868*	4.319*	2.902	1.766	0.824
	Health status	3.010	1.716	1.052	0.544	1.871	0.953	0.953	1.714
Living characteristics	Living duration	1.413	2.288	2.443	0.102	8.363*	2.562	1.085	5.173
	Living status	0.793	0.793	0.013	0.667	0.024	0.024	0.024	0.000
	Housing choice	0.190	2.337	4.107	6.109*	3.023	0.964	1.120	2.510
Economic characteristics	Monthly income	6.232	15.266**	12.549*	12.106*	4.666	9.748*	13.308**	8.560
	Whether paying social insurance	0.757	3.636	3.498	4.570	2.213	1.506	2.213	1.321
Individual attitude characteristics	Sense of gain	5.827	6.695	7.447	8.981*	4.533	8.919*	10.366*	10.452*
	Sense of safety	9.412*	11.043*	7.296	7.583	7.581	8.555	7.886	10.222*
	Sense of happiness	5.792	5.061	4.581	4.028	4.923	4.147	6.822	5.829
	Perception of community services	8.776	8.966*	11.034*	9.537*	9.569*	8.466	11.556*	10.931*
	Desire for smart community services	14.099**	11.700*	15.662**	15.732**	16.150**	13.881**	10.753*	15.948***

1. The data in the table are chi-square values; 2. *** $p < 0.001$, ** $p < 0.01$, * $p < 0.05$
