# Supplementary material for: Demands for Community Services and Associated Factors among Residents in Smart Communities: A Case Study of Xuzhou City
Source: Int J Environ Res Public Health. 2023 Feb 20;20(4):3750. doi: 10.3390/ijerph20043750 (PMC9964050; doi:10.3390/ijerph20043750)
Supplement: Supplementary file 1 [file ijerph-20-03750-s001.zip › Supplementary File S5.pdf]

Table S4. Binary logistic analysis results of demands for community services in smart communities with a demand proportion of more than 80%.

Category	Type	SFM	SEGS	ESND	ESA	ESPHE	TS	SRS	FSE
Sociodemographic characteristics	Gender (Female)	0.503	0.739	0.606	0.632	0.546	0.814	0.681	1.256
	Age	1.226	1.237	1.288	1.161	1.406	1.528	1.295	1.142
	Career	1.000	1.000	1.000	1.000	1.000	1.000	1.000	1.000
	Staff of state-owned enterprises and institutions	1.075	0.468	1.398	0.748	1.002	1.576	2.069	0.909
	Staff of private and foreign enterprises and institutions	0.909	0.856	1.950	0.555	1.289	1.370	2.568	1.340
	Individual industrial and commercial household	1.499	1.013	1.735	1.076	4.515	2.758	5.756	3.916
	Freelancer	1.706	0.654	1.239	1.186	2.784	2.292	6.235	3.525
	Student	0.561	0.160	2.826	0.724	2.963	2.927	6.922	6.889
	Other	0.588	0.339	0.802	0.754	1.447	3.491	3.937	3.112
	Educational level	1.140	1.029	1.206	1.057	1.246	1.451*	1.402*	1.537*
	Marital status (Unmarried)	2.128	5.207	0.665	1.195	0.604	0.625	0.994	0.477
Living characteristics	Living duration	0.766	0.773	0.818	0.684	0.792	0.779	1.100	0.612
	Housing choice	1.000	1.000	1.000	1.000	1.000	1.000	1.000	1.000
	House owner	1.195	1.413	2.061	2.050	1.655	0.904	0.975	0.440
	Other	1.011	0.660	2.577	0.639	1.062	0.507	0.603	0.265
Economic characteristics	Monthly income	1.197	1.486*	1.344	1.289	1.075	1.384	1.542*	1.403
	Whether paying social insurance	1.066	0.948	0.914	0.794	0.780	1.862	0.807	0.836

Category	Type	SFM	SEGS	ESND	ESA	ESPHE	TS	SRS	FSE
Individual attitude characteristics	Sense of gain	1.006	1.615	0.803	1.527	1.211	1.103	1.653	1.887
	Sense of safety	0.833	0.592	0.957	0.559	0.654	1.022	0.634	0.606
	Perception of community services	1.541	1.458	1.614*	1.595	1.399	1.188	1.528	1.146
	Desire for smart community services	0.432**	0.523*	0.470**	0.483**	0.528*	0.619	0.627	0.832
The *p* value of Hosmer and Lemeshow Test		0.359	0.197	0.144	0.543	0.341	0.620	0.110	0.346
Percentage of correct prediction		83.3%	81.9%	81.0%	81.9%	83.3%	81.0%	82.4%	86.0%

1. The data in the table are OR values; 2. *** $p < 0.001$, ** $p < 0.01$, * $p < 0.05$
