# Supplementary material for: Demands for Community Services and Associated Factors among Residents in Smart Communities: A Case Study of Xuzhou City
Source: Int J Environ Res Public Health. 2023 Feb 20;20(4):3750. doi: 10.3390/ijerph20043750 (PMC9964050; doi:10.3390/ijerph20043750)
Supplement: Supplementary file 1 [file ijerph-20-03750-s001.zip › Supplementary File S3.pdf]

Questionnaire on demands of residents for community services in smart communities

Dear Sir/Madam,

Hello, we are very pleased to invite you to participate in our study. Our research team is currently conducting academic research related to residents' demands in the smart community. In order to explore residents' demands for community services in smart communities and clarify its influencing factors, we hope that you can spare a little time to finish this anonymous questionnaire. We will keep it strictly confidential, and thank you for your participation!

Section 1: Basic Information

1. What is the name of the community you live in?

____

2. What's your gender?

A. Male

B. Female

3. What is your age?

A. 17 years old and below

B. 18-35 years old

C. 36-45 years old

D. 46-69 years old

E. 70 years old and above

4. What's your occupation now? (or before retirement)

A. Civil servant

B. Staff of state-owned enterprises and institutions

C. Staff of private and foreign enterprises and institutions

D. Individual industrial and commercial household

E. Freelancer

F. Student

G. Other

5. What is your educational level?

A. Primary school or below

B. Middle school

C. High school and technical secondary school

D. Junior college

E. Bachelor degree

F. Master's degree or above

6. What is your marital status?

A. Married

B. Unmarried

7. What is your health condition?

A. Good

B. General

C. Bad

8. How long you have lived in your community?

A. Less than 1 year

B. 1 to 3 years

C. More than 3 years

9. How do you live?

A. Living alone

B. Not living alone

10. What is your housing choice?

A. Renter

B. House owner

C. Other

11. What is your disposable income for a month?

A. Within 1000 RMB (about 146.69 USD)

B. 1000-3000 RMB (about 146.69-440.09 USD)

C. 3000-5000 RMB (about 440.09-733.54 USD)

D. 5000-7000 RMB (about 733.54-1026.92 USD)

E. Above 7000 RMB (about 1026.92 USD)

12. Do you pay social insurance?

A. All

B. Partly (e.g., only medical insurance)

C. Not at all

13. Do you think that your current community life is full of a sense of gain? (Sense of gain: A positive psychological feeling of comfort, belonging, and happiness that is subjectively produced by community residents since the rights, interests, and demands of community residents in life, work, study, entertainment, and other aspects are guaranteed and satisfied because of the various services provided by the community using the new generation of information technology in the new era of smart communities.)

A. Couldn’t agree more

B. Agree

C. Generally

D. Disagree

E. Couldn’t disagree more

14. Do you think that your current community life is full of a sense of safety? (Sense of safety: The subject evaluation and inner feelings that affect the individual survival and development demands at present and for some time to come.)

A. Couldn’t agree more

B. Agree

C. Generally

D. Disagree

E. Couldn’t disagree more

15. Do you think that your current community life is full of a sense of happiness? (Sense of happiness: The overall judgment of residents on the quality of life of smart communities in the new era, including the degree of satisfaction with the content of smart community construction in the new era and the resulting psychological state of positive emotion dominance, which is the organic unity of subjective well-being and

psychological well-being.)

A. Couldn’t agree more

B. Agree

C. Generally

D. Disagree

E. Couldn’t disagree more

16. What is the degree of your perception of community services?

A. Very well

B. Well

C. General

D. Not well

E. Very little

17. Do you yearn for smart community services?

A. Absolutely

B. A little

C. Generally

D. Unlikely

E. Not at all

Section 2: Demands for community services in smart communities

18. Which of the following community services in smart communities do you need? (Tick the box if you need this service)

**Category**	**Type**	**Demand**
Smart business service	1. Self-service Supermarket (SS)	
	2. Smart E-commerce System (SES)	
	3. Smart Farmers Market (SFM)	
	4. Package Delivery System (PDS)	
	5. Smart Childcare System (SCS)	
	6. Service Platform for House Cleaning (SPHC)	
Smart property service	1. Smart Management System of Water and Electricity (SMSWE)	
	2. Smart Illumination System in Public Area (SISPA)	
	3. Smart Parking System (SPS1)	

Category	Type	Demand
	4. Smart Security System of Property Management (SSSPM)	
	5. Smart Payment System (SPS2)	
	6. Smart Waste Bin (SWB)	
	7. Maintenance Management System of Property Management (MMSPM)	
	8. Smart Environmental Greening System (SEGS)	
Smart emergency service	1. Emergency System of Natural Disaster (ESND)	
	2. Emergency System of Accident (ESA)	
	3. Emergency System of Social Security Event (ESSSE)	
	4. Emergency System of Public Health Event (ESPHE)	
Smart medical care service	1. Smart Healthcare Service Center (SHSC)	
	2. Smart Medical Record System (SMRS)	
	3. Telemedicine System (TS)	
	4. Smart Referral System (SRS)	
Smart elderly care service	1. Smart Elderly Care Facilities (SECF)	
	2. Smart Elderly Health Examination (SEHE)	
	3. Online Lectures about Elderly Care (OLEC)	
	4. Health Record Management System of the Elderly (HRMSE)	
	5. Appointment System of Regular and Free Medical Consultations for the Elderly (ASRFMCE)	
	6. First-aid Service for the Elderly (FSE)	
Smart communication service	1. Smart Forum (SF)	
	2. Smart Activity Center (SAC)	
	3. Psychological Counseling (PC)	
Smart government service	1. Demand Expression Platform (DEP)	
	2. Government Service System (GSS)	
	3. Grid Management System (GMS)	
	4. Employment Platform (EP)	
	5. Legal Service (LS)	
	6. Smart Bulletin Board (SBB)	
	7. Poverty Assistance (PA)	
	8. Volunteer Service System (VSS)	
